# Cryogenic 3D Printing of *ß*-TCP/PLGA Composite Scaffolds Incorporated With BpV (Pic) for Treating Early Avascular Necrosis of Femoral Head

**DOI:** 10.3389/fbioe.2021.748151

**Published:** 2022-01-18

**Authors:** Feng Li, Zhifu Cao, Kai Li, Ke Huang, Chengliang Yang, Ye Li, Chuanchuan Zheng, Yulu Ye, Tingjie Zhou, Haoqiang Peng, Jia Liu, Chong Wang, Kegong Xie, Yujin Tang, Liqiang Wang

**Affiliations:** ^1^ Department of Orthopaedics, Affiliated Hospital of Youjiang Medical University for Nationalities, Baise, China; ^2^ Youjiang Medical University for Nationalities, Baise, China; ^3^ The Third Affiliated Hospital of Southern Medical University, Guangzhou, China; ^4^ Guangxi Key Laboratory of basic and translational research of Bone and Joint Degenerative Diseases, Baise, China; ^5^ Guangxi Biomedical Materials Engineering Research Center for Bone and Joint Degenerative Diseases, Baise, China; ^6^ School of Mechanical Engineering, Dongguan University of Technology, Dongguan, China; ^7^ State Key Laboratory of Metal Matrix Composites, School of Material Science and Engineering, Shanghai Jiao Tong University, Shanghai, China

**Keywords:** bisperoxovanadium, *ß*-tricalcium phosphate, avascular necrosis of femoral head, phosphatase and tensin homolo, rats

## Abstract

Avascular necrosis of femoral head (ANFH) is a disease that is characterized by structural changes and collapse of the femoral head. The exact causes of ANFH are not yet clear, but small advances in etiopathogenesis, diagnosis and treatment are achieved. In this study, *ß*-tricalcium phosphate/poly lactic-co-glycolic acid composite scaffolds incorporated with bisperoxovanadium [bpV (pic)] (bPTCP) was fabricated through cryogenic 3D printing and were utilized to treat rat models with early ANFH, which were constructed by alcohol gavage for 6 months. The physical properties of bPTCP scaffolds and *in vitro* bpV (pic) release from the scaffolds were assessed. It was found that the sustained release of bpV (pic) promoted osteogenic differentiation and inhibited adipose differentiation of bone marrow-derived mesenchymal stem cells. Micro-computed tomography scanning and histological analysis confirmed that the progression of ANFH in rats was notably alleviated in bPTCP scaffolds. Moreover, it was noted that the bPTCP scaffolds inhibited phosphatase and tensin homolog and activated the mechanistic target of rapamycin signaling. The autophagy induced by bPTCP scaffolds could partially prevent apoptosis, promote osteogenesis and angiogenesis, and hence eventually prevent the progression of ANFH, suggesting that the bPTCP scaffold are promising candidate to treat ANFH.

## Introduction

Avascular necrosis of femoral head (ANFH) is a disease that is related to necrosis of bone cells and marrow tissue, structural changes and collapse of the femoral head, joint dysfunction and pain (Seijas et al., 2017). ANFH is a slow-course disease with complex etiology and a high disability rate, leading to the loss of a patient’s physical labor ability (Zalavras and Lieberman, 2014). It is known that alcohol is an important pathogenic factor in the pathogenesis of ANFH, and among Chinese male ANFH patients, 40.4% of the patients had alcoholism (Moya-Angeler, 2015). Since most people in China have the custom of drinking a high dose of alcohol for a long time, the incidence of ANFH has been increasing year by year (Cui et al., 2015). The most effective surgical approach for ANFH is total hip arthroplasty (THA), but due to the limited life-time of the artificial joints, it is still controversial (Scaglione, 2015). Therefore, developing a novel treatment strategy for ANFH is of great importance and demand.

In the last decade, scaffold-based bone tissue engineering has gained increasing attention in various orthopaedic applications (Sadiasa et al., 2013; Tao et al., 2019; Guo et al., 2020). It is a key scientific issue to prevent and cure ANFH by implanting bioactive materials combined with biologically active factors of key regulatory pathways (Sharma et al., 2019). Biocompatible and biodegradable poly (lactic-co-glycolic acid) (PLGA) is the most widely used synthetic polymer to encapsulate drugs/biomolecules (Thi Hiep et al., 2017; Swider et al., 2018). The addition of TCP to PLGA could improve the mechanical properties of PLGA and reduce the risk of inflammation by neutralizing the acidic products due to the hydrolysis of PLGA matrix.

Phosphatase and tensin homolog deleted on chromosome ten (PTEN), which has a phosphatidylinositol 3-phosphatase activity, is a very effective negative regulator of the P13K/Akt pathway. Akt and its downstream target proteins play a crucial role in the regulation of bone formation and remodeling (Liu et al., 2007; Mukherjee and Rotwein, 2009). The enhanced activity of PTEN can inhibit the activity of mechanistic target of rapamycin (mTOR) pathway (Yang et al., 2018). mTOR pathway is an evolutionary conserved signaling that regulates cell proliferation, autophagy, and apoptosis by participating in multiple signaling pathways. Studies have shown that the mTOR pathway is associated with cancer, arthritis, insulin resistance, osteoporosis and other diseases. Bisperoxovanadium [bpV (pic)] is an inhibitor of PTEN, which binds to the active center and inhibits the activity of PTEN protein (Schmid et al., 2004). *In vivo* and *in vitro* experiments have proved that bpV (pic) has a strong inhibitory effect on PTEN at a low concentration. Targeted inhibition of PTEN can increase the content of Alkaline phosphate (ALP) in osteoblasts, improve mineral formation, and up-regulate the expression of genes related to bone formation (Liu et al., 2017), suggesting bpV (pic) is an excellent drug candidate to treat bone degeneration diseases by inhibiting PTEN activity.

In this study, we fabricated bpV (pic)/TCP/PLGA porous scaffolds (designated as “bPTCP”) through cryogenic 3D printing to treat early avascular necrosis of femoral head models in rats, which was made through a 6-month alcohol gavage to rats. The scaffold used in this experiment was porous, and its unique porous pore structure could provide sufficient contact area for the growth of bone tissue cells and blood vessels. The average porosity of cortical bone in rats is 10%–20%, while the porosity of bone trabecular bone is between 50% and 90% (Smith et al., 2020). We noticed that the bPTCP scaffolds promoted osteogenic differentiation and inhibited adipose differentiation in cultured bone Mesenchymal Stem Cells (BMSCs). (Bone Mesenchymal Stem Cells). Furthermore, bPTCP scaffolds induced autophagy, partially prevented apoptosis, promoted osteogenesis and angiogenesis and prevented the progression of ANFH. The underlying mechanism is that controlled release of bpV (pic) from bPTCP scaffolds inhibited PTEN and activated mTOR signalings. In short, the fabricated bPTCP scaffolds could be a promising therapeutic strategy to prevent ANFH.

## Materials and Methods

### Materials

PLGA with a molecular weight of 100,000 was provided by Sigma Aldrich (United States). bpV (pic) and *ß*-TCP powder (with a diamter <10 μm) were provided by Shanghai Tissue Engineering Center (China). Methylene dichloride (DCM) was provided by Shanghai Clinical Research Center (China). Deionized (DI) water was prepared by an ultra-pure water system (Arum 611, Sartorius).

### Scaffold Fabrication

**FIGURE 1:**
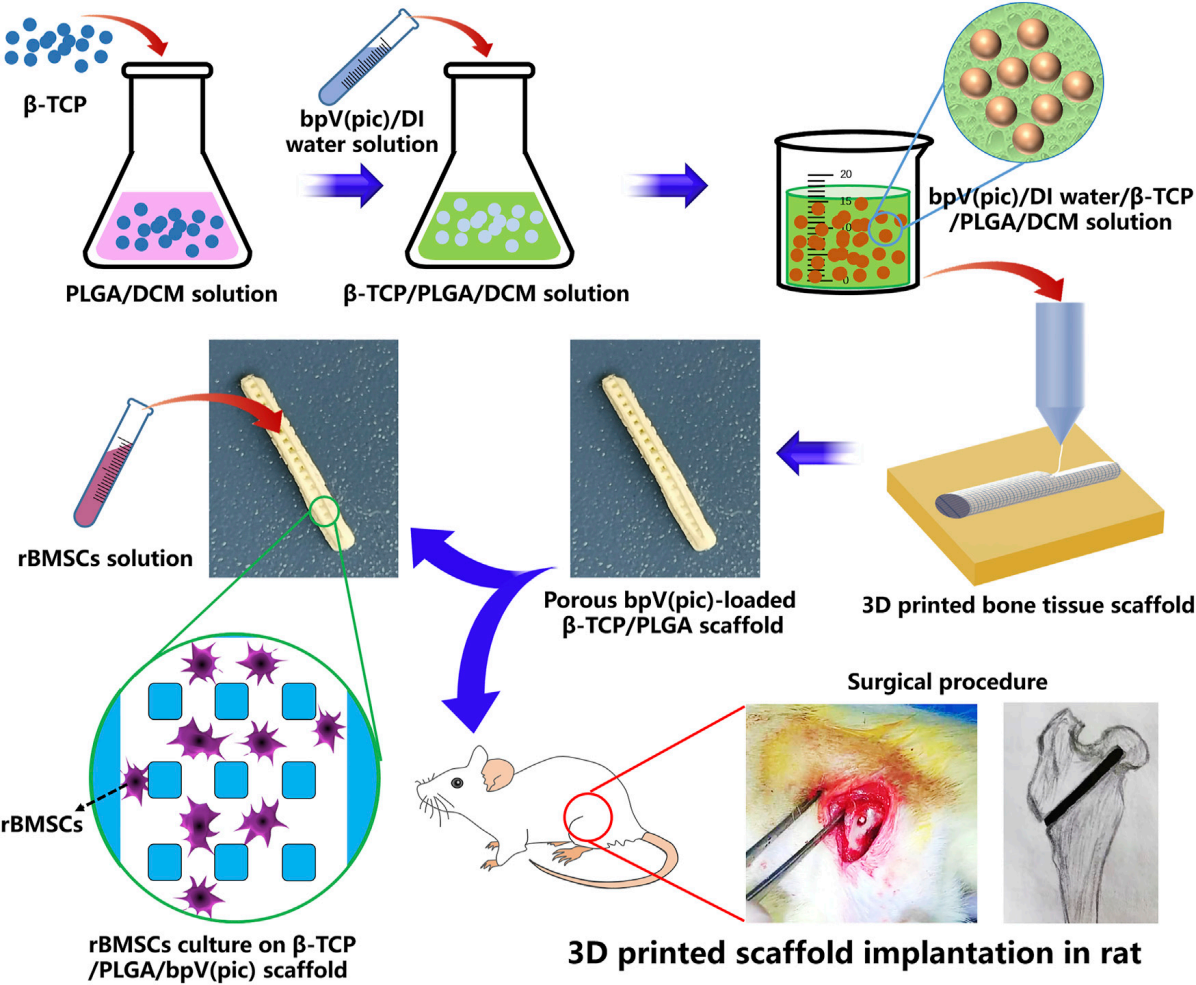
Schematic illustration of fabricating bpV (pic)/TCP/PLGA composite scaffolds *via* cryogenic 3D printing. The scaffolds were seeded with rBMSCs for *in vitro* biological evaluate and implanted into the femoral heads of rats with ANFH to treat ANFH *in vivo*.

The formulation of printing inks and the fabrication process of the 3D printed bone tissue engineering scaffolds (designated as “CTP”) are shown in Figure 1. Firstly, 4 g of PLGA was dissolved in 10 ml of DCM, followed by the addition of 3 g of TCP particles. With the assistance of 30 min of ultra-sonication in an ice water bath, uniform TCP/PLGA/DCM suspension was obtained. Subsequently, we added 2 ml of DI water containing 25 μg of bpV (pic) into the TCP/PLGA/DCM suspension, followed by the addition of 100 μL of Tween20 as emulcifier. In our study, Tween 20 was solely used as a biocompatible surfactant to stabilize the as-formulated composite emulsion inks. It has been used in many studies and no cell toxicity was reported (Wang et al., 2017). With the aid of sufficient oscillation, water-in-oil composite emulsions was obtained. The as-prepared composite emulsions were used as printing inks and poured in a 20 ml syringe which was connected with a V-shape nozzle (inner diameter of 0.4 mm). The ink loaded syringe was further loaded in the cryogenic 3D printer which could provide a cryogenic environment with a temperature of −30 C. The propelling speed of the ink was maintained at 0.002 ml/s, and the printing speed is set at 8 mm/s. A pre-designed STL file was imported in the cryogenic 3D printer and 3D scaffolds with a rod-like pattern were fabricated. Scaffolds were first dried in a freeze-dryer for 24 h and then coated with a thin layer of gold. The coated scaffold samples were attached to the stage and subjected to scanning electron microscopy (SEM) observation (Sigma 500, Zeiss, Germany). Compression tests were conducted to study the mechanical properties of scaffolds using a universal testing machine (Shanghai He Sheng Instrument co., ltd).

### Scaffold Characterization

Digital camera and SEM were used to observe the macro- and micro-structures of different scaffolds. Typically, scaffold samples with a dimension of 5 mm × 5 mm × 5 mm were immersed in PBS solution and then subjected to a compression testing using a universal testing machine at 37°C. The strain rate was set as 2 mm/min and the compression was stopped when 50% compression strain was achieved. Five samples for each type of scaffold were tested. The compression strength and elastic modulus were calculated.

### Alcoholic Femoral Head Necrosis Model of Rats

Healthy adult SD rats were provided by The Experimental Animal Center of Youjiang Medical University for Nationalities, and all procedures were approved by the Animal Ethics Committee of our hospital. According to the experimental method of Wang et al. (2019) and Zhang et al. (2016), the experimental animals were changed from rabbits to rats. A total of 60 2-month-old healthy SD male rats with a bodyweight of 260–310 g (285 g in average) were selected. Rats have free access to water and food (temperature: 22 ± 1°C; relative humidity: 45 ± 5%). Automatic control of light and shade period is about 12 h. After adaptive feeding for 1 week, 10 rats were selected as negative control (sham group), 50 rats were gavaged with 52.0% alcohol liquor at 0.01 ml/g for 4 months. The 50 rats were examined by X-ray, *µ*-CT and MRI after 4 months of intragastric administration, and 42 animals were confirmed to suffer from ANFH. 30 rats in good status were selected and randomly divided into control group (ANFH group), PLGA-TCP stent group (PTCP group) and bpV (pic)-PLGA-TCP stent group (bPTCP group) (*n* = 10). The rats were anesthetized by intraperitoneal injection of 3% pentobarbital sodium (1 ml/kg body weight), and the left femoral trochanter was exposed. The pulp core was decompressed with a 1.2 mm surgical drill, and a bone tunnel with a diameter of 1.2 mm was established under the guidance of C-arm fluoroscopy. Two different stents were placed in the bone tunnel, sutured one after the other, the antibiotic used within the scope of animal experiments is penicillin. After the surgery, the rats were intramuscularly injected with penicillin at a dose of 1.0 × 106 U/kg for three consecutive days. Two months later, the animals were sacrificed and bone tissues of femoral heads from all the groups were analyzed.

### Fourier Transform Infrared Spectra Analysis

Fourier transform infrared (FTIR) spectra of two scaffolds were recorded with a NICOLET iS5 (Thermo fisher, United States) FTIR spectrometer using a KBr pellet technique with a resolution of 1 cm^−1^ over a scan range of 4,000–500 cm^−1^.

### Degradation Behaviour Experiment

The *in vitro* degradation behaviour of scaffolds was studied by monitoring the weight remaining in an 8-week test period. Typically, 100 mg of scaffold sample was put into a 15 ml centrifuge tube which was added with 3 ml PBS solution. The test liquid was changed every week. After 2-, 4-, 6-, and 8-weeks of incubation, the scaffold samples were taken out and rinsed in DI water for several times to remove precipitated salts. The rinsed scaffolds were then freeze-dried for 48 h and the weight remaining was measured using a digital balance.

### Cell Culture

Bone marrow mesenchymal stem cells (BMSCs) were derived from the femoral cavity of adult rats. 10% fetal bovine serum was added into Dulbecco’s Modified Eagle Medium (DMEM)-high sugar medium, and rat BMSCs cell was inoculated into the Medium (Derubeis and Cancedda, 2004). They were then cultured in a cell incubator at 37°C, CO2 concentration was 5% and relative humidity was 50%. The medium was changed timely according to the growth rate of cells, and the cells with 80–90% confluence were digested with 0.25% EDTA working solution of trypsin for a subculture or cell seeding. To further study the osteogenesis capability of scaffolds, scaffold samples were infused with 0.5 ml of rat bone marrow-derived mesenchymal stem cells (rBMSCs) solution with a density of 5 × 107 cell/mL. In order to achieve the adhesion of stem cells to the bone tissue structure scaffold, the scaffolds and rBMSCs were cultured together *in vitro*, and the effects of inhibiting PTEN activation of mTORC1 on the mTORC1 activity and osteogenic differentiation function of rBMSCs were detected. BpV concentration was determined by referring to the cell culture method previously studied (Mukhopadhyay et al., 2014), The cells were stimulated with leaching solution extracted from bPTCP scaffolds (two concentrations) and the scaffold was infiltrated with 5 ml (bpV-1) or 10 ml (bpV-2) culture medium for 48 h. The purpose of using different concentrations of bpV was to study whether different concentrations would affect the behavior of cells.

### Alkaline Phosphate Staining

BMSCs in different groups (control, bpV-1, bpV-2) were cultured with mineralization-inducing media containing 100 µM ascorbic acid (Sigma-Aldrich, St. Louis, MO. United States), 2 mM *ß*-glycerophosphate (Sigma-Aldrich) and 10 nM dexamethasone (Sigma-Aldrich). For ALP staining, after induction, cells were fixed with 70% ethanol and incubated with a solution of 0.25% naphthol AS-BI phosphate and 0.75% Fast Blue BB dissolved in 0.1 M Tris buffer (pH 9.3). ALP activity assay was performed with an ALP kit according to the manufacturer’s protocol (Sigma-Aldrich) and normalized based on protein concentrations.

### Oil Red O Staining

BMSCs in different groups (control, bpV-1, bpV-2) were cultured with medium containing a-MEM, 10% FBS, 100 U/ml penicillin, 100 mg/ml streptomycin, 5 mg/ml insulin (Sigma-Aldrich), 1 mM dexamethasone (Sigma-Aldrich), 0.5 mM 3-isobutyl-1-methylxanthine (Sigma-Aldrich), and 100 mM indomethacin (Sigma-Aldrich) to induce adipogenic differentiation. For Oil red O staining, cells were washed twice with PBS and fixed with 4% paraformaldehyde for 2 h at 4°C. Then, cells were stained for 2 h in freshly diluted oil red O solution at 4°C. The stain of cells was removed by washing twice with PBS.

### Histological, Immunofluorescence and TUNEL Analysis

Resected bone samples from each group were fixed in 4% paraformaldehyde and decalcification with 10% EDTA for 2 months. After decalcification, bone samples were cut into 5-μm-thick sections and stained with hematoxylin and eosin (H and E) following a standard protocol. For immunofluorescence analysis, the slices were incubated in 10 mM citric acid buffer overnight at 60°C to unmask antigens. Then, we incubated the slices in diluted primary antibodies at 4°C overnight and appropriate secondary antibody for 1 h at RT. nuclei were counterstained in 4′,6-diamidino-2-phenylindole (DAPI) (Life Technologies) and images were obtained on a confocal laser-scanning microscope (Olympus, Tokyo, Japan). For TdT-mediated dUTP nick end labeling (TUNEL), slices were deparaffinized and antigens were unmasked. The DeadEnd Fluorometric TUNEL System (Promega) procedure was performed following the manufacturer’s instructions.

### Western Blot Analysis

The cells were lysed in 2% sodium dodecyl sulfate (SDS), 2 M urea, 10% glycerol, 10 mM Tris-HCl (pH 6.8), 10 mM dithiothreitol, and 1 mM phenylmethylsulfonyl fluoride. Proteins were separated by 10% SDS-polyacrylamide gel electrophoresis. After electrophoresis, the proteins were transferred to the membrane by wet transfer (Bio-Rad Laboratories, Hercules, CA, United States). Each membrane was incubated with TBST (100 mM Tris-HCl pH 7.5, 150 mM NaCl, 0.05% Tween 20) and 5% non-fat-blocking milk powder at room temperature for 1 h, then incubated overnight with the primary antibody in a shaking bottle at 4°C. The membrane and HRB-conjugated secondary antibody were incubated at room temperature for 1 h. The membrane was then treated with enhanced chemiluminescence reagent (ECL Kit, Amersham Biosciences, Piscataway, NJ, United States) and the proteins were detected using chemiluminescence technology.

### Real-Time Polymerase Chain Reaction Analysis

Expression of osteogenic genes, including alkaline phosphate (ALP), collagen type I (col1a1), osteocalcin (OCN), transcription factor Sp7 (Osterix), and Runt-related transcription factor 2 (RUNX2), were analyzed in BMSCs in different groups with osteogenic induction medium. And adipogenesis genes including Fatty acid-binding protein 4 (Ap2), CCAAT/enhancer-binding protein *a* (CEBP*α*), peroxisome proliferator-activated receptor γ (PPARγ) and adiponectin were analyzed. Operation steps: 1) Measure the concentration of total RNA extracted, separate the mRNA, and then use the reverse transcription kit for operation, reverse transcription of the mRNA into cDNA. 2) The purpose of PCR primers is to design a pair of appropriate nucleotide fragments that can effectively amplify template DNA sequences. After culturing for 7 days, total RNA was extracted using Trizol reagent from cells. The concentration of RNA was measured by a NanoDrop spectrophotometer (Thermo Fisher Scientific, United States). The primers used are shown in Table 1.

**TABLE 1:** Primer sequences used for RT-PCR.

Genes	Primer sequences
ALP	forward: 5′-CGG ATC CTG ACC AAA AAC C-3′
	reverse: 5′-TCA TGA TGT CCG TGG TCA AT-3′
col1a1	forward: 5′-CTG ACC TTC CTG CGC CTG ATG TCC-3′
	reverse: 5′-GTC TGG GGC ACC AAC GTC CAA GGG-3′
OCN	forward: 5′-CAC CAT GAG GAC CCT CTC TC-3′
	reverse: 5′-TGG ACA TGA AGG CTT TGT CA-3′
Osterix	forward: 5′-TCT CCA TCT GCC TGA CTC CT-3′
	reverse: 5′-AGC GTA TGG CTT TGT GC-3′
Runx2	forward: 5′-GAC TGT GGT TAC CGT CAT GGC-3′
	reverse: 5′-ACT TGG TTT TTC ATA ACA GCG GA-3′
aP2	forward: 5′-ATG GGA TGG AAA ATC AAC CA-3′
	reverse: 5′-GTG GAA GTG ACG CCT TTC AT-3′
C/EBP*α*	forward:5′-CAC CTG CAG TTC CAG ATC G-3′
	reverse: 5′-GTA CTC GTT GCT GTT CTT GTC CAC-3′
PPAR*γ*	forward: 5′-AGA CAT TCC ATT CAC AAG AAC AGA-3′
	reverse: 5′-TGA ACT CCA TAG TGA AAT CCA GAA-3′
adiponectin	forward:5′-TTG GTC CTA AGG GAG ACA CG-3′
	reverse: 5′-CAC ACT GAA TGC TGA GCG GTA-3′
GAPDH	forward:5′-CAT GTA CGT TGC TAT CCA GGC-3′
	reverse: 5′-CTC CTT AAT GTC ACG CAC GAT-3′

### Micro-CT Scanning

Micro-computed tomography (*μ*-CT, Scanco Medical, Bassersdorf, Switzerland) was used to determine rats’ femoral heads from all groups (voltage 75 kV, resolution 12 μm per pixel). The bone mineral density (BMD) and bone volume/total volume (BV/TV) were calculated with the analysis system of the micro-CT. A total of 100 sections of the primary trabecular bone of the lower femoral metaphysis as areas of interest were quantified.

### Statistical Analysis

All data were expressed as mean ± standard deviation (SD). SPSS statistical software (version 16.0) was used for one-way ANOVA (*p* < 0.05), and the difference was statistically significant.

## Results and Discussion

### Scaffold Characterization

**FIGURE 2:**
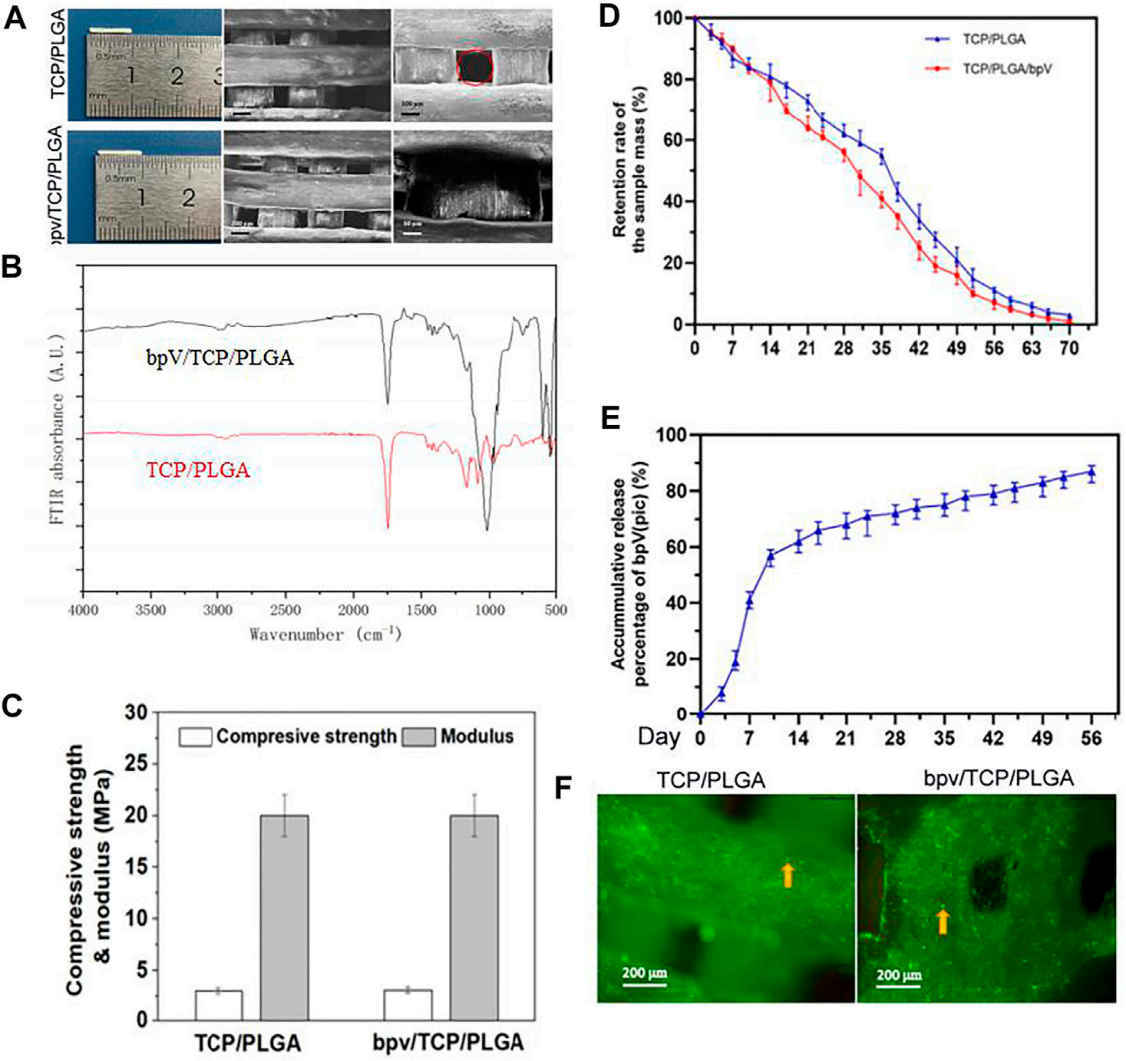
Morphology and mechanical properties of TCP/PLGA and bpV/TCP/PLGA scaffolds. **(A)** The morphology of the 3D-printed PTCP and bPTCP scaffolds and SEM micrographs of different scaffolds at different magnification; **(B)** FTIR spectra of the 3D-printed PTCP and bPTCP scaffolds. **(C)** Compressive strengths and elastic modulus of both scaffolds. **(D)** *In vitro* degradation behavior of both scaffolds. **(E)** *In vitro* release behavior of bPTCP scaffolds in a 8-week test period. **(F)** Results of inverted fluorescence microscope observation of cultured cells for 3 days, the round dots in the figure were living cells (yellow arrow).

The general morphology and different magnification under SEM of the PTCP and bPTCP scaffolds is shown in Figure 2A. The scaffolds had a rod-like structure in which grid patterns can be observed. The strut surface was rough. and all scaffolds showed a good dispersion of TCP particles. Figure 2B showed the FTIR of TCP/PLGA before and after bpV (pic) incorporation. It can be seen that the distinctive peaks at 1,631 and 805 cm−1 were corresponding to the–OH stretching vibrations of TCP/PLGA, intimating that there is–OH group existing on the surface of the scaffolds. The distinctive peaks at 1,000 cm-1belonged to the C-H flexural vibration of the olefin group and alkyl group. The adsorption peak at 547 cm−1 presented the P-O stretching vibration of phosphate group, and the adsorption peak at 1,629 cm−1 belonged to the C = O stretching vibration of the carboxyl group. After bpV (pic) adsorption, the peak at 805 cm−1 was shifted to 801 cm−1, indicating the hydrogen bonds were formed between the TCP/PLGA and bpV (pic). Similarly, the C-H, C-O, C = O and–OH peaks area of TCP/PLGA tended to a lower peak area and the vibration band at 1,238 cm−1 presented the C=N stretching bands, The vibration band at 1,045 cm−1 presented the C-N stretching bands, revealing that there was an incorporation between the TCP/PLGA material and bpV (pic). Indicating that bpV was successfully loaded on the bPTCP scaffold. The both compressive strength of two scaffolds are 2.8 MPa, and the both elastic moduli are 20.0 MPa. There were no significant differences in pore size, porosity and mechanical properties in each scaffold group. The addition of bpV (pic) did not change the mechanical properties (Figure 2C). The *in vitro* degradation behavior of different scaffolds was studied by monitoring their weight remaining in test liquid within 10 weeks (Figure 2D). The degradation rate of PTCP stent was slightly slower than bPTCP, and the degradation rate of bPTCP was more than 96% in the 10th week. The *in vitro* release behavior of bPTCP scaffold was also studied (Figure 2E). 60% level of bpV (pic) was released within 10 days, and the release profile achieved a plateau after 14 days of incubation. The release of bpV (pic) reached 87% in the 8th week at a nearly constant rate. After scaffold characterization, we studied the biological performance of scaffolds. rBMSCs were cultured on the scaffolds for 3 days and live and dead staining was conducted. It is noticed that the viability of rhBMSCs on different scaffolds had no significant difference (Figure 2F), indicating that the two scaffolds have similar biocompatibility to cells and that the 3D printed scaffolds were biocompatible platforms for BMSCs growth.

### bPTCP Scaffolds Promotes Osteogenic Differentiation of Rats BMSCs

**FIGURE 3:**
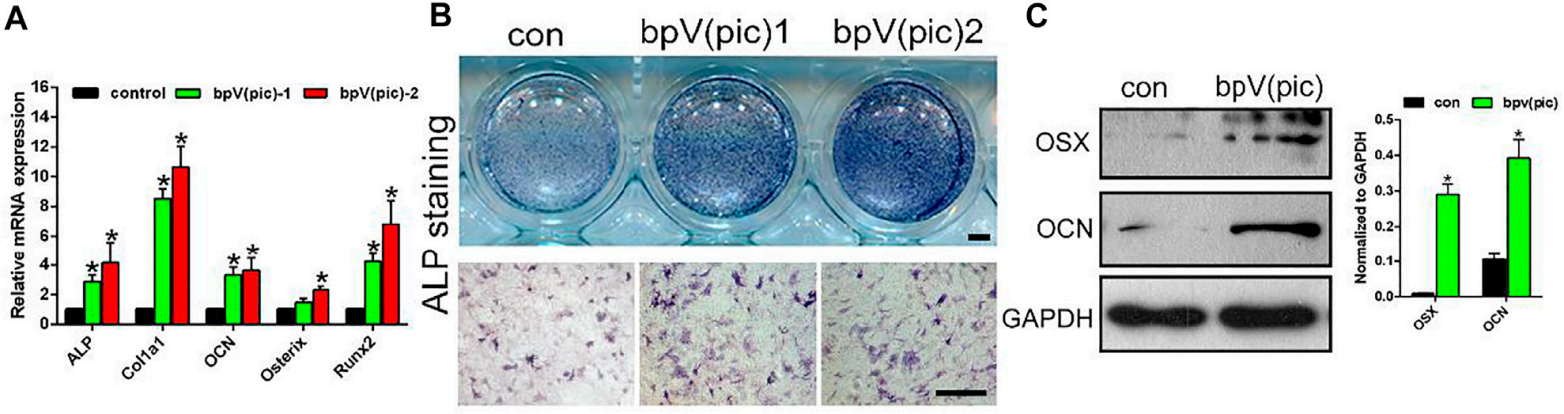
bPTCP scaffolds promotes osteogenic differentiation of rats BMSCs. **(A)** mRNA expression of osteogenic genes in BMSCs stimulated with osteogenic medium supplemented control or two different concentrations of extracts from the bPTCP scaffolds [bpV (pic)-1, bpV (pic)-2] for 7 days. B. ALP staining of the BMSCs stimulated with osteogenic medium supplemented control or two different concentrations of extracts from the bPTCP scaffold for 7 days. C. Western blot results of OSX and OCN in BMSCs stimulated with osteogenic medium supplemented control or extracts from the bPTCP scaffold for 7 days *p < 0.05.

rBMSCs have multi-lineage differentiation potential, including the ability to form osteoblasts, adipocytes and chondrocytes. To analyze the effect of bPTCP scaffolds on the rBMSC osteogenesis, we cultured the rBMSCs with osteogenic medium supplemented with two types of extracts from the scaffolds (different concentrations). The mRNA expressions of the osteogenic differentiation-related genes were analyzed with RT-PCR, and we noticed that the gene expression level of ALP, Col1*α*1, OCN, Osterix and Runx2 were significantly up-regulated in the treatment of extracts from the bPTCP scaffolds (Figure 3A). Also, the ALP activity was enhanced in the bPTCP groups compared with control group (Figure 3B). We confirmed that the protein expression of osterix and OCN were up-regulated in the stimulation of bPTCP scaffolds (Figure 3C). These data indicated that bPTCP scaffolds promote the osteogenic differentiation of BMSCs.

### bPTCP Scaffolds Inhibited Adipogenic Differentiation of Rats BMSCs

**FIGURE 4:**
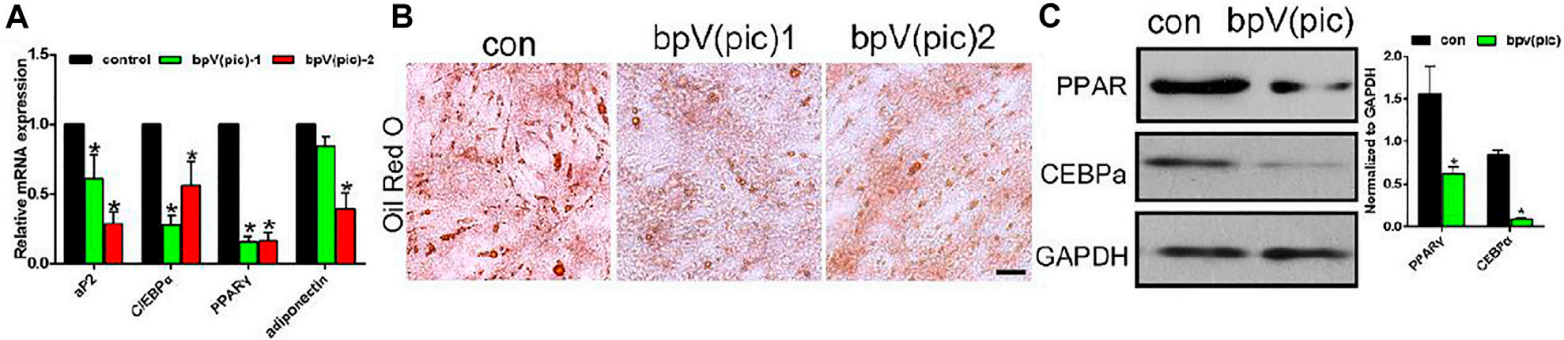
bPTCP scaffolds inhibited adipogenic differentiation of rats BMSCs. **(A)** mRNA expression of adipogenesis genes in BMSCs stimulated with adipogenic medium supplemented control or two different concentrations of extracts from the bPTCP scaffold for 7 days. **(B)** Oil red O staining of BMSCs stimulated with adipogenic medium supplemented control or two different concentrations of extracts from the bPTCP scaffold for 7 days. **(C)** Western blot results of PPAR*γ* and CEBP*α* in BMSCs stimulate with adipogenic medium supplemented control or extracts from the bPTCP scaffold for 7 days *p < 0.05.

Next, we evaluated the effect of bPTCP scaffolds on the adipogenic differentiation of rats BMSCs. In the stimulation of adipogenic medium and extracts from the bPTCP scaffolds, the mRNA expression of adipocyte differentiation genes were all decreased, including Ap2, C/EBPα, PPARγ and adiponectin (Figure 4A). The lipid production in the differentiated adipocytes was also inhibited in the bPTCP group, as shown by oil red O staining (Figure 4B). Western blotting analysis confirmed that the protein level of PPAR*γ* and C/EBP*α* were down-regulated in the bPTCP group (Figure 4C). In summary, these data suggest that bPTCP scaffolds inhibit the adipogenic differentiation of rats BMSCs.

### bPTCP Scaffolds Induced Autophagy and Inhibited Apoptosis *via* Activating AKT/mTOR Signaling in rBMSCs

**FIGURE 5:**
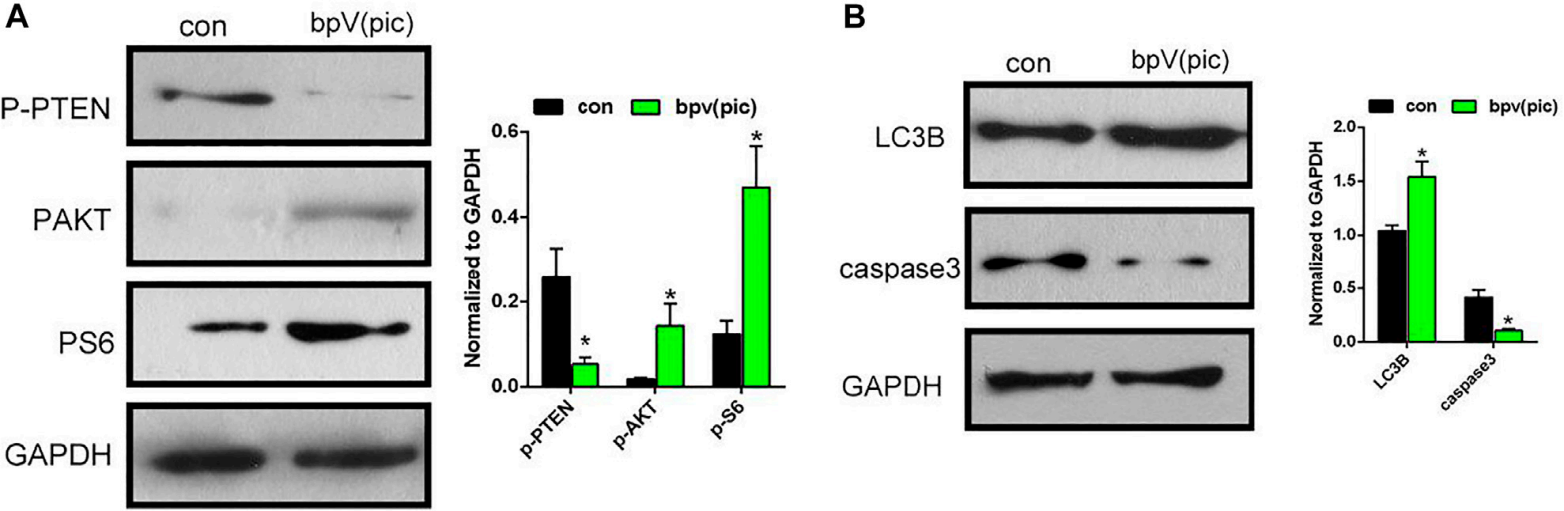
bPTCP scaffolds induced autophagy and inhibited apoptosis via activating AKT/mTOR signaling *in vitro.* **(A)** Western blot results of *p*-PTEN, *p*-AKT, p-S6 in BMSCs stimulate with control or extracts from the bPTCP scaffold for 24 h. **(B)** Western blot results of autophagy and apoptosis marker (LC3B and cleaved-caspase3) in BMSCs stimulate with control or extracts from the bPTCP scaffold for 24 h.

Previous studies had reported that inhibition of PTEN by bpV (pic) would lead to the activation of AKT/mTOR signaling. The loss of PTEN function leads to AKT/mTOR activation, and subsequently increased osteogenic differentiation in osteoblasts. Therefore, we investigated whether the bPTCP scaffolds work through the inhibition of PTEN by controlled release of bpV (pic). We evaluated the expression of phosphorylation state of PTEN and downstream proteins of AKT/mTOR signaling in BMSCs with western blot analysis. As shown in Figure 5A, expression of phosphorylated-PTEN was down-regulated in bPTCP group, indicating the activity of PTEN was inhibited by the controlled release of bpV (pic). Moreover, expression of phosphorylated AKT and S6 levels were both up-regulated, which means the activation of AKT/mTOR signaling in the treatment of bPTCP scaffolds. Furthermore, we noticed the elevated expression of autophagy and reduced apoptosis related protein in the treatment of bPTCP scaffolds (Figure 5B). Overall, our data confirm that bPTCP scaffolds work through the controlled release of bpV (pic). Inhibition of PTEN by bPTCP scaffolds activates AKT/mTOR signaling, induces autophagy and inhibits apoptosis in cultured BMSCs.

### bPTCP Scaffolds Alleviated the Progression of ANFH in Rats

**FIGURE 6:**
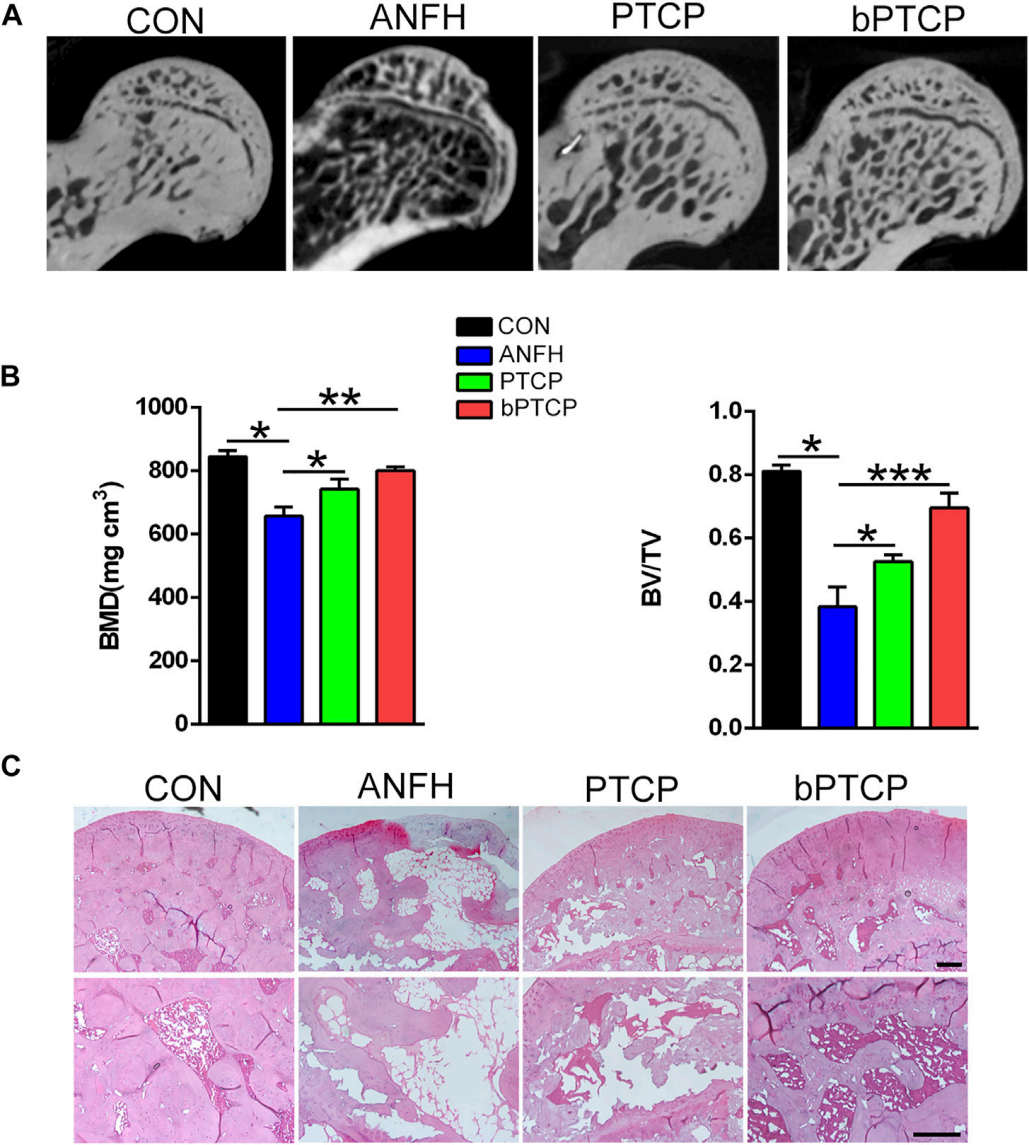
bPTCP scaffolds alleviated the progression of ANFH in rats. **(A)** Micro-CT scanning images of femoral heads from ANFH rats with different treatments. **(B)** Quantification of the value of BMD and BV/TV in both groups; **(C)** H and E staining of the femoral head in ANFH rats from different groups. *n* = 10 for each group. Scale bar = 50 μm, *p < 0.05. * *p < 0.01, * **p < 0.001.

To elucidate the role of bPTCP scaffolds in the progression of ANFH, we established an ANFH rat model and implanted the PTCP control and bPTCP scaffolds into the left femoral trochanter of ANFH rats. The bone tissues of femoral heads from all the groups were collected and analyzed at 2-month post-surgery. Micro-CT scanning confirmed the presence of significantly fewer subchondral trabeculae in the ANFH group compared to the normal group, and PTCP treated rats showed a limited increase while the bPTCP group showed a more significant increase in the number of trabeculae (Figure 6A). The quantification of BMD and BV/TV verified that the bPTCP group had a better effect in the alleviation of ANFH progression compared to other groups (Figure 6B). Next, the femoral heads from all groups were embedded and sectioned. H and E staining showed increased numbers of empty lacunae and cell necrosis in the subchondral trabecular area of ANFH group, rats in bPTCP group showed decreased numbers of empty lacunae compared to ANFH and PTCP group (Figure 6C). These data indicate that bPTCP scaffolds alleviate the progression of ANFH in rats.

### bPTCP Scaffolds Prevented ANFH by Inducing Autophagy and Inhibiting Apoptosis *in vivo*

**FIGURE 7:**
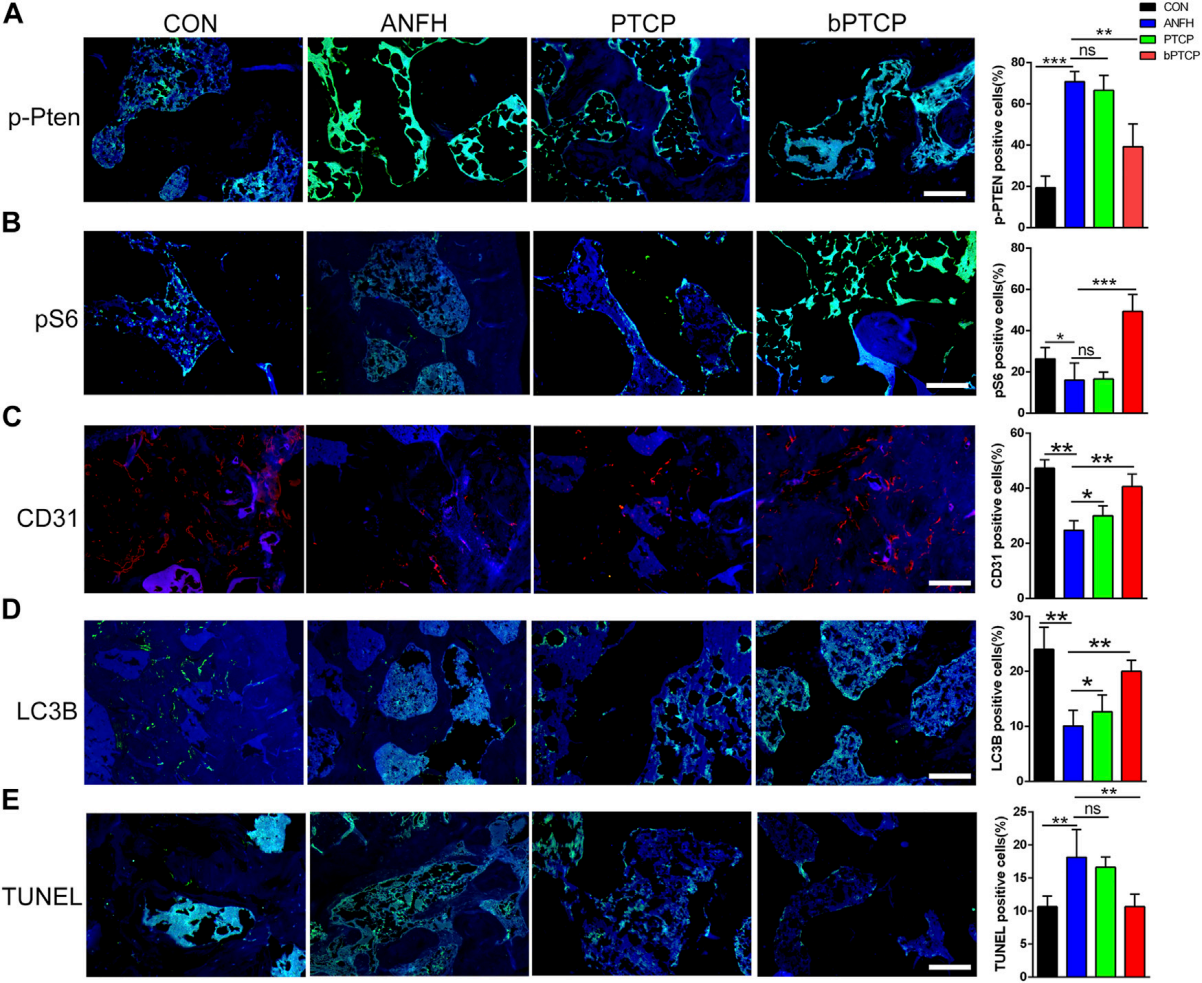
bPTCP scaffolds prevented ANFH by inducing autophagy and inhibiting apoptosis *in vivo.* Representative images of IF staining of *p*-PTEN **(A)**, p-S6 **(B)**, CD31 **(C)** LC3B **(D)** and TUNEL **(E)** in the femoral head of ANFH rats from different groups. ns, not significant, *p < 0.05. * *p < 0.01, * **p < 0.001.

Finally, we examined the underlying mechanism of bPTCP scaffolds in alleviating ANFH *in vivo*. With IF staining, we noticed that the expression of *p*-PTEN was notably enhanced in the bone section of ANFH rats, and treated with bPTCP scaffolds decreased *p*-PTEN expression while PTCP cannot (Figure 7A). Consistent with the *in vitro* study, rats treated with bPTCP scaffolds also activated mTOR signaling as expression of p-S6 elevated in bPTCP group compared with others (Figure 7B). Next, with IF staining, we confirmed the increased level of angiogenic marker CD31 (Figure 7C) and autophagy marker LC3B (Figure 7D) in bPTCP group. Moreover, with TUNEL analysis to label the apoptosis cells in bone sections, we noticed a reversed effect of bPTCP scaffolds on the increased apoptosis in ANFH rats. In conclusion, our data demonstrate that bPTCP scaffolds could promote angiogenesis and prevent ANFH, and the underlying mechanism is through activating AKT/mTOR signaling, inducing autophagy and inhibiting apoptosis.

To date, ANFH is still a great challenge to orthopedic surgeons as the exact mechanisms remain unclear. It is characterized by bone ischemia and destruction of the microstructure, resulting in increased adipose vacuoles and collapse of the femoral head (Pierce et al., 2019; Wu et al., 2019). BMSCs are capable to differentiate into various cell types including osteoblasts, adipocytes, myoblasts and chondrocytes (Liu R. et al., 2019; Zheng et al., 2020). The imbalance of osteoblastic and adipogenic differentiation swift of BMSCs correlates with some diseases including osteoporosis and ANFH (Liu J. et al., 2019).

PTEN is a tumor suppressor gene on chromosome 10. This protein can counteract the effect of PI3K and terminate some downstream signaling pathways. In addition, AKT may be the main downstream target of PTEN, and experiments have confirmed that bpV (pic) has a strong inhibitory effect on PTEN *in vivo* and *in vitro* (Schmid et al., 2004). The PI3K/AKT pathway regulates the activation of mTORC1 by regulating the phosphorylation of Rheb, the upstream direct activator of mTORC1 (Aoki and Fujishita, 2017). Therefore, PTEN can reduce the expression level of the downstream effector phosphorylated ribosomal protein S6 (S6) of mTORC1 by inhibiting PI3K/AKT/mTORC1 (Wang et al., 2021). Our *in vitro* experiments showed that under bpV (pic) intervention, the expression of PTEN protein was inhibited, and the expression levels of AKT and S6 protein were significantly up-regulated, further indicating that bpV (pic) can inhibit the expression of PTEN, and then activate the PI3K/AKT/mTORC1 pathway, which is up-regulated in the expression of corresponding indicators and osteogenic factor OCN. The high expression of Osx suggests that bpV (pic) can promote the osteogenesis of rBMSCs cells to a certain extent by activating the PI3K/AKT/mTORC1 pathway. CD31 is an important marker of angiogenesis, and its staining in the TCP/PLGA/bpV group was significantly stronger than that in the TCP/PLGA/bpV group and THE ANFH group, indicating that bpV (pic) not only has an osteogenic effect, but also has the potential to promote angiogenesis. In the process of osteogenesis, angiogenesis can also promote osteogenesis, which can accelerate bone formation and promote the increase of bone mass (Zhang et al., 2020).

Micro-ct analysis results show TCP/PLGA group The implant in the TCP/PLGA/bpV (pic) group was degraded 12 weeks after surgery, so CT images of the implanted stent could not be presented. This phenomenon was consistent with the results of previous studies, but it could be seen that the damaged bone trabecular was repaired, and the scaffold material in the TCP/PLGA group was completely degraded, indicating TCP/PLGA biomaterial have good degradability and can be degraded within 12 weeks, which is conducive to our observation of changes in the influence of new bone. We can exclude the influence of scaffold materials on bone mass measurement results. BMD in TCP/PLGA/bpV (pic) group BV/TV value was higher than that of TCP/PLGA group and ANFH group. Three-dimensional imaging of bone trabecular growth in TCP/PLGA group was better than that in the ANFH group, but worse than that in TCP/PLGA/bpV(PIC) group. It can be seen that the tunnel cavity of the ANFH group was filled with sparse and limited bone trabeculae, and the bone trabeculae in the femoral head were sparse, and the number and thickness of bone trabeculae in the ANFH group were significantly lower than those in the TCP/PLGA/bpV group. In the TCP/PLGA group, the trabecular area of the bearing area was rare, and the normal trabecular microstructure was lost, so it was difficult to maintain the shape and size of the femoral head, and it was easy to form femoral head collapse and progress to irreversible femoral head necrosis (Zou et al., 2015). By comparing the bone mass indexes of each group, it was clear that the trabecular area of the femoral head was strengthened in the TCP/PLGA/bpV group.

As an inhibitor of PTEN activity, the effect of bpV (pic) has been discussed in spinal cord injury, traumatic brain injury, stroke, and other neurological disease models (Shi et al., 2011; Liu R. et al., 2019; Liu J. et al., 2019; Yu et al., 2020). The mechanistic influences of bpV (pic) activity including activating PI3K/AKT/mTOR, ERK1/2 and *ß*-Catenin signaling (Sury et al., 2011; Liu et al., 2018; Tang et al., 2019; Walker et al., 2019). In our study, we noticed that released bpV (pic) from bPTCP scaffolds promoted osteogenic differentiation and inhibited adipose differentiation in cultured BMSCs. Consistent with the results of previous studies, we found that bPTCP scaffolds inhibited PTEN activity to activate the AKT/mTOR signaling, therefore promoted autophagy and inhibited apoptosis. Autophagy and apoptosis are two important processes of programmed cell death (Mukhopadhyay et al., 2014). Previous studies have shown that osteoblast apoptosis increased and autophagy reduced in the early phase of ANFH (Zheng et al., 2020). Thus, we speculated that bpV (pic) could be a good candidate drug for ANFH.

However, intraperitoneal injection of bpV (pic) aqueous solution is the most conventional way of drug administration, but bpV (pic) is unstable in water which makes it difficult to accomplish *in vivo* studies. Further, the repair of osteonecrosis regeneration takes a long time, and the efficiency of the drug reaching the target site is low. Therefore, it is particularly important to prepare a sustained release system of bpV (pic) to directly and continuously work on the ANFH site. PLGA is a biodegradable material approved by the U.S. Food and Drug Administration (FDA) for use as a continuous release vector for medical materials, drugs, and cytokines. *ß*-TCP ceramics also owns good biocompatibility and similarity, and has been widely used matrix for bone repairment (Xie et al., 2015). Therefore, we constructed bpV (pic) composite scaffolds and loaded them into PLGA with *ß*-TCP stents and applied them into the rat’s model of ANFH.

In addition to osteogenesis, angiogenesis is also critical to the survival of newly formed bone and bone regeneration in ANFH (Li et al., 2013; Liu et al., 2018). With the implantation of bPTCP scaffolds, the present study demonstrated osteogenesis and angiogenesis were both enhanced in the ANFH rats. The increased bone growth was in accordance with the *in vitro* study, but the limitation of this study is that we did not discuss the mechanism of bpV (pic) on the angiogenesis.

## Conclusion

In this study, to treat alcohol-induced AFNH, we successfully fabricated TCP/PLGA composite scaffolds incorporated with bpV (pic) via cryogenic 3D printing. It is highly consistent with the porosity of trabecular bone of rats, indicating that the 3D printed bone tissue scaffold has a mechanical structure that highly fits the cancellous bone of rats. Released bpV (pic) from the bPTCP scaffolds promoted osteogenic differentiation and retarded adipogenic differentiation of rBMSCs *in vitro*. bPTCP scaffolds activated Akt/mTOR signal by inhibiting PTEN activity and hence promoted autophagy and inhibited cell apoptosis. In addition, we noticed that bPTCP scaffolds increased vascular remodeling that stimulate necrotic tissue and improve blood circulation, thereby promoting new bone formation and preventing ANFH progression.

## Data Availability

The original contributions presented in the study are included in the article/supplementary material, further inquiries can be directed to the corresponding authors.
